# Physiological and autonomic stress responses after prolonged sleep restriction and subsequent recovery sleep in healthy young men

**DOI:** 10.1007/s41105-017-0122-x

**Published:** 2017-09-08

**Authors:** Wessel M. A. van Leeuwen, Mikael Sallinen, Jussi Virkkala, Harri Lindholm, Ari Hirvonen, Christer Hublin, Tarja Porkka-Heiskanen, Mikko Härmä

**Affiliations:** 1 Department of Physiology, Medicum, University of Helsinki, PO Box 63, 00014 Helsinki, Finland; 2 Centre of Expertise for the Development of Work and Organizations, Finnish Institute of Occupational Health, Topeliuksenkatu 41 a A 00250 Helsinki, Finland; 3 Division for Sleep and Alertness Research, Stress Research Institute, Stockholm University, 10691 Stockholm, Sweden; 4 Agora Centre, University of Jyväskylä, PO Box 35, 40014 Jyväskylä, Finland; 5 Department of Clinical Neurophysiology, Medical Imaging Centre, Pirkanmaa Hospital District, PO Box 2000, 33521 Tampere, Finland; 6 Centre of Expertise for Health and Work Ability, Finnish Institute of Occupational Health, Topeliuksenkatu 41 a A 00250 Helsinki, Finland

**Keywords:** Sleep restriction, Autonomic nervous system, HPA-axis, Cortisol, Heart rate variability

## Abstract

**Purpose:**

Sleep restriction is increasingly common and associated with the development of health problems. We investigated how the neuroendocrine stress systems respond to prolonged sleep restriction and subsequent recovery sleep in healthy young men.

**Methods:**

After two baseline (BL) nights of 8 h time in bed (TIB), TIB was restricted to 4 h per night for five nights (sleep restriction, SR, *n* = 15), followed by three recovery nights (REC) of 8 h TIB, representing a busy workweek and a recovery weekend. The control group (*n* = 8) had 8 h TIB throughout the experiment. A variety of autonomic cardiovascular parameters, together with salivary neuropeptide Y (NPY) and cortisol levels, were assessed.

**Results:**

In the control group, none of the parameters changed. In the experimental group, heart rate increased from 60 ± 1.8 beats per minute (bpm) at BL, to 63 ± 1.1 bpm after SR and further to 65 ± 1.8 bpm after REC. In addition, whole day low-frequency to-high frequency (LF/HF) power ratio of heart rate variability increased from 4.6 ± 0.4 at BL to 6.0 ± 0.6 after SR. Other parameters, including salivary NPY and cortisol levels, remained unaffected.

**Conclusions:**

Increased heart rate and LF/HF power ratio are early signs of an increased sympathetic activity after prolonged sleep restriction. To reliably interpret the clinical significance of these early signs of physiological stress, a follow-up study would be needed to evaluate if the stress responses escalate and lead to more unfavourable reactions, such as elevated blood pressure and a subsequent elevated risk for cardiovascular health problems.

## Introduction

The restorative function of sleep in many of our bodily systems, including the immune and cardiovascular systems, has been widely documented [1–5]. Despite the clear beneficial functions of sleep, voluntary sleep restriction is getting increasingly common in modern industrialized societies, due to, for instance, increasing access to electronic entertainment at times that were originally devoted to sleep [6]. In addition, atypical and excessive working hours contribute to sleep restriction in the working population [7–9]. Finally, restricted sleep is a common phenomenon among those suffering from certain psychiatric or physical disorders [10].

The consequences of insufficient sleep are severe and widespread [11]. First of all, it results in sleepiness that contributes to increasing amounts of traffic and work-related accidents [12–15]. Sleep restriction also has adverse effects on mood and cognitive performance [16–19].

Probably the most serious direct effects, however, are those on health and bodily systems. Within the immune system, for instance, inflammatory markers rise in response to restricted sleep making it a risk factor for cardiovascular diseases [20–23]. It has also been shown that short sleep per se is associated with an increased mortality risk [24, 25].

Hence, it is important to unravel the mechanisms through which sleep and health are related to find ways and strategies to help people with chronically restricted sleep and patients with sleep disorders.

Stress systems allow us to adapt to a changing and/or challenging situation to maximize the likelihood of survival. Acute total sleep loss is an example of such a situation and it is therefore not surprising that the activity of the two main neuroendocrine systems of the integrated stress response (i.e., the autonomic sympatho-adrenal system and the hypothalamic pituitary–adrenal axis) responds.

Although many studies have investigated how acute total sleep loss affects stress systems, the results are far from consistent. Several studies have, for instance, failed to show an effect of total sleep loss on cortisol profiles [26–29], whereas others point towards an increase in cortisol levels [30, 31]. Studies on the effects of acute total sleep loss on heart rate show also inconsistent results. Some studies point toward increased heart rate in response to total sleep loss [32], others to a decrease [33] and yet others to no effect at all [34].

Autonomic nervous system activity, as measured by heart rate variability (HRV), has been shown to change toward increased sympathetic activity after total sleep loss [32, 33, 35, 36]. Pagani et al. however, could not confirm such a shift toward increased sympathetic activity [34].

Much fewer studies have been carried out adopting a prolonged partial sleep loss design. Pejovic and colleagues did not observe changes in cortisol levels after six nights of 6 h sleep [37], whereas for instance Spiegel and colleagues have reported increased evening cortisol levels after six nights of 4 h sleep, but only when compared with a 12 h sleep extension condition [38]. Also regarding blood pressure, previous studies are far from consistent. Dettoni et al. found all hemodynamic parameters to be unaffected after five nights of restricted sleep [39], whereas Tochikubo et al. have reported an increase in blood pressure after a night with restricted sleep [40].

Concerning HRV effects, some studies point toward an increase in sympathetic activity [39, 41], whereas others failed to show such an increase [38]. None of these studies, however, have adopted a design that reflects a situation that could occur during a working week.

Since prolonged partial sleep restriction is very common in working life, it is vital to fully understand how such a condition affects our stress systems. As described above, previous knowledge is not consistent. Therefore, in the present study, we simulated accumulating sleep restriction during five working days followed by 2 days of weekend recovery sleep and measured the changes in several parameters of both the autonomic sympatho-adrenal system and the hypothalamic pituitary–adrenal (HPA) axis that occurred during this period. Previously, we have revealed that this sleep restriction design disturbs immune and metabolic functions as well as cognitive task performance and self-perception of cognitive performance [19, 22, 42, 43]. Hence, we here hypothesize (1) increased activity in both components of the integrated stress response and (2) increased sympathetic and decreased parasympathetic modulation in line with the results of Dettoni et al. [39].

## Materials and methods

### Participants and design

23 healthy men, aged 19–29 years (mean ± SD 23.1 ± 2.5 years), participated in this study and were recruited during a 2-year time span. After a telephone interview, participants underwent a thorough physical examination including blood tests (triglycerides, cholesterol, haemoglobin, creatinine, leukocytes, erythrocytes, hematocrit, TSH, ASAT, ALAT, MCV, MCH, and MCHC) and screening polysomnography. Final eligibility was evaluated according to pre-determined inclusion and exclusion criteria, as described earlier [43]. Two weeks prior to the experiment, participants completed sleep diaries, had an adaptation night in the sleep laboratory, and carried actigraphs to verify adherence to a regular sleep–wake schedule (i.e., sleep between 2300 and 0700 hours). The pre-study actigraphy-based mean sleep duration (±SD) was 6.88 (±0.58) h in the control group and 7.05 (±0.80) h in the experimental group.

The study design was approved by the ethics committee of Helsinki University Central Hospital and written informed consent was obtained from participants. The experiment was conducted at the Brain and Work Research Centre of the Finnish Institute of Occupational Health.

**Fig. 1:**
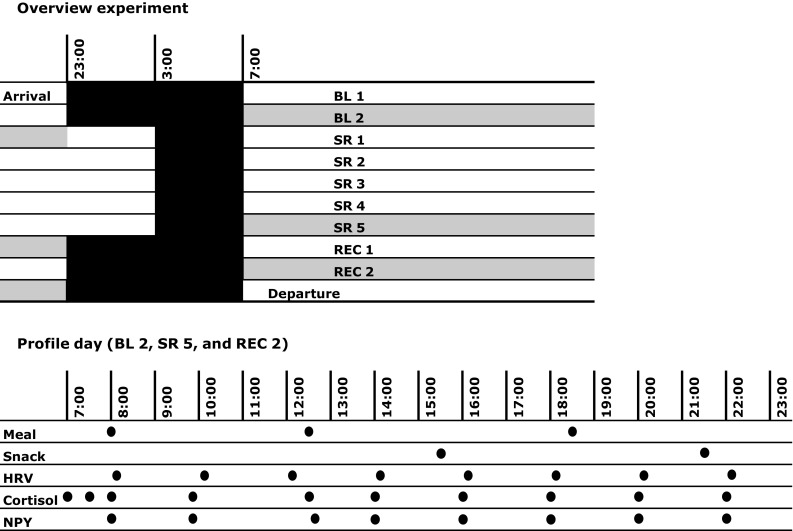
The experimental protocol. After two nights of 8 h TIB (baseline, BL), TIB was restricted to 4 h per night for five subsequent nights (sleep restriction, SR), followed by three nights of 8 h TIB (recovery, REC). Profile days where measurements took place are shaded in gray. The lower panel shows a profile day in more detail

15 participants, who were randomly allocated to the experimental group (EXP), spent the first two nights 8 h in bed (baseline, BL; from 2300 to 0700 hours), followed by five nights of 4 h in bed (sleep restriction, SR; from 0300 to 0700 hours), and, finally, again three nights of 8 h in bed (recovery, REC; Fig. 1). The remaining eight participants served as the control group (CON) and spent 8 h in bed every night. Sleep during daytime was not allowed, which was monitored by continuous EEG recordings and a continuously present investigator. During waking, participants took part in a bigger experiment of our sleep laboratory, involving the simulation of a working week by a variety of cognitive and psychological tasks, also during the nights that were subject to restricted sleep.

Participants ate standardized meals at fixed times throughout the experiment: breakfast at 0800 hours (600 kcal), lunch at 1230 hours (800 kcal), dinner at 1830 hours (700 kcal), and snacks at 1530 hours (300 kcal) and 2130 hours (200 kcal). In addition, participants in EXP ate a piece of fruit (apple or orange) at 0030 hours (50 kcal). Participants were not allowed to leave the building, but could, during regular short breaks, leave the sleep and test room and visit a spare time room with a television and a personal computer.

Illumination in the sleep and test room ranged, at all times, from 150 to 400 L and in the spare time room from 350 to 600 L The temperature ranged from 19 to 23 °C. Caffeine consumption was neither allowed during the experiment nor 1 week before. Non-caloric fluid intake (e.g., water) was not subject to any limitations.

### Heart rate, blood pressure, and cardiac autonomic control measurements

The cardiac autonomic control was evaluated by 10 min recordings at supine rest between 0800 and 0900 hours. During this test period, interbeat intervals of cardiac function were registered continuously by the electrocardiogram (ECG, WinAcq, Absolute Aliens, Finland) and the peripheral blood pressure was monitored by photopletysmographic method (Portapres, Finapres Medical Systems, The Netherlands). The circulatory parameters were calculated with special software of neurocardiological analyses (WinCPRS, Absolute Aliens, Finland) [44, 45].

The following parameters were assessed directly: RR intervals (RRIs), heart rate, systolic blood pressure, diastolic blood pressure, mean arterial pressure and pulse pressure. Stroke volume and cardiac output were calculated from these directly assessed data. In addition, the following components included in measuring short-term spectral analysis (FFT method) of HRV and blood pressure variability: total power, ultralow-frequency (ULF, <0.0033 Hz) power, very low-frequency (VLF, 0.0033 to <0.04 Hz) power, low-frequency (LF, 0.04 to <0.15 Hz) power, and high-frequency (HF, 0.15 to <0.40 Hz) power.

### Heart rate variability measurements

Whole day HRV measurements were done on the three profile days indicated in Fig. 1. On these days, HRV was determined during 5-min epochs that were selected about every 2 h (i.e., at approximately 0800, 1000, 1200, 1400, 1600, 1800, 2000, and 2200 hours) when subjects were sitting during the rest periods between the cognitive task sessions. Ambulatory ECG was recorded at 200 Hz and analyzed using Somnologica Studio Software (Version 3.3.1, Medcare, Reykjavik, Iceland). RRIs during the epochs were detected by Somnologica and checked for artifact correction and rejection according to established standards [45, 46] and stored in an RRI ASCII file. From the RRI file, power spectral density (PSD) was calculated, using Kubios HRV Software 1.16 (University of Kuopio, Kuopio, Finland), with the default parametric autoregressive model. Powers were calculated for different frequency bands: very low frequency (VLF, 0–0.04 Hz), low frequency (LF, 0.04–0.15 Hz), and high frequency (HF, 0.15–0.40 Hz) in absolute units (ms2), normalized units (n.u.) and as percentage of total power. In addition, the ratio between the LF and HF bands was calculated.

### Salivary neuropeptide Y and cortisol assays

Saliva samples for the analysis of NPY levels were collected 8 times a day on the three profile days (Fig. 1): at 0800, 0950, 1240, 1400, 1600, 1800, 2000, and 2200 h. NPY levels were measured using a commercial EIA immunoassay kit [Phoenix Pharmaceuticals, Inc; Neuropeptide Y (NPY); human, rat]. The measurement range was 0.10–100 ng/mL (linear range 0.1–1.4 ng/mL) with assay repeatability values of 5% (within series) and 8% (between series). These results are not absolute concentrations of NPY because of matrix effects (i.e., effects of other substances in the sample), but the samples of one participant at the three profile days are comparable.

Saliva samples for the analysis of cortisol levels were collected ten times a day on the three profile days (Fig. 1): at 0700, 0730, 0800, 0950, 1230, 1400, 1600, 1800, 2000, and 2200 hours. Cortisol levels were measured using a commercial kit assay (Salivary Cortisol, LIA, IBL, Hamburg, Germany). The measurement range was 0.43–110 nmol/L with assay repeatability values of 5% (within series) and 8% (between series).

### Statistical analysis

HRV, NPY, and cortisol levels were analyzed using repeated measures ANOVA to test for main effects of day, time, and day × time. For cortisol, due to the strong well-known morning peak, this was done separately for morning values (*t* ≤ 1230 hours) and for afternoon/evening values (*t* > 1230 hours). The other data that were measured only once per profile day were also analyzed using repeated measures ANOVA and tested for an effect of day. A *p* value <0.05 was considered to be statistically significant. For cortisol, outliers (>2 × SD from mean, *n* = 13) and missing values (*n* = 2) were replaced by the group mean value for that specific time point. Group (EXP or CON) was not included as factor in the ANOVA, due to highly unequal group sizes and consequently unequal standard deviations.

All statistical analyses were carried out using SPSS Statistics 19.0.0 for Mac OS X (IBM Corporation, New York, USA) and the figures were generated using GraphPad Prism 5.0 for Mac OS X (GraphPad Software Inc., La Jolla, USA).

## Results

### Effectiveness of the protocol

To ensure the effectiveness of the sleep restriction protocol, participants rated their sleepiness levels using the Karolinska Sleepiness Scale (KSS) [47] 14 times on every test day. In EXP, the daily mean KSS score went from 4.3 ± 0.3 at BL, to 6.2 ± 0.4 at SR, to 3.8 ± 0.4 at REC [*F*(2,24) = 21.98, *p* < .001]. In CON, the daily mean KSS scores did not change throughout the experiment.

### Cardiovascular parameters

**Fig. 2:**
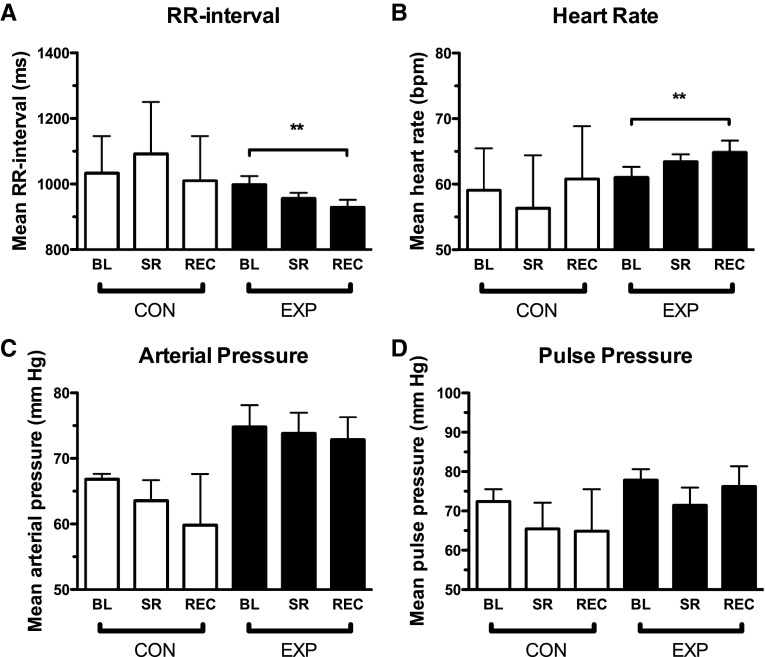
RR interval (**a**), heart rate (**b**), arterial pressure (**c**), and pulse pressure (**d**) at baseline (BL), and after sleep restriction (SR) and recovery (REC) in the control group (CON) and the experimental group (EXP). Values are means ± SEM, **p < .01

The results of the cardiovascular parameters are shown in Table 1 and Fig. 2. In CON, none of the parameters changed during the experiment and in EXP only heart rate rose significantly during the experiment (Fig. 2). The RRI declined from 1009 ± 27 ms at BL to 967 ± 15 ms at SR to 929 ± 23 ms at REC [*F*(2,22) = 9.89, *p* = .001]. In terms of beats per minute, this meant an increase from 60 ± 1.8 bpm at BL to 63 ± 1.1 bpm at SR to 65 ± 1.8 bpm at REC [*F*(2,18) = 6.29, *p* = .008]. All other parameters, including blood pressure, remained unaffected (Table 1).

**Table 1:** Basic cardiovascular parameters results

	Control group				Experimental group			
	BL	SR	*REC*	*p*	BL	SR	REC	*p*
RRI interval (ms)	1033 ± 112	1092 ± 158	1010 ± 135	*F* = 3.41, *p* = .227	1009 ± 27	967 ± 15	929 ± 23	*F* = 9.89, *p* = .001
Heart rate (bpm)	59 ± 6.3	56 ± 8.1	61 ± 8.0	*F* = 5.08, *p* = .164	60 ± 1.8	63 ± 1.1	65 ± 1.8	*F* = 6.29, *p* = .008
Systolic arterial pressure (mmHg)	120 ± 3.4	111 ± 8.5	107 ± 17	*F* = 1.03, *p* = .491	132 ± 5.0	127 ± 5.9	130 ± 6.6	*F* = 0.431, *p* = .655
Diastolic arterial pressure (mmHg)	48 ± 0.35	45 ± 1.8	42 ± 6.0	*F* = 0.940, *p* = .515	55 ± 3.3	55 ± 3.4	54 ± 2.9	*F* = 0.135, *p* = .874
Mean arterial pressure (mmHg)	67 ± 0.80	64 ± 3.1	60 ± 7.8	*F* = 0.965, *p* = .509	74 ± 3.5	74 ± 3.4	73 ± 3.4	*F* = 0.083, *p* = .921
Pulse pressure (mmHg)	72 ± 3.1	65 ± 6.7	65 ± 11	*F* = 1.23, *p* = .448	77 ± 2.9	72 ± 5.3	76 ± 5.2	*F* = 0.727, *p* = .496
Stroke volume (ml)	90 ± 7.2	86 ± 3.3	86 ± 2.7	*F* = 1.06, *p* = .486	84 ± 2.5	80 ± 2.5	84 ± 2.8	*F* = 1.20, *p* = .321
Cardiac output (L/min)	5.4 ± 0.98	4.9 ± 0.87	5.2 ± 0.84	*F* = 12.06, *p* = .077	5.1 ± 0.24	5.0 ± 0.15	5.5 ± 0.15	*F* = 2.47, *p* = .107

Values are means ± SE. *F* and *p* values indicate results of repeated measurements ANOVA. For abbreviations, refer to the text

### Short-term spectral analyses of blood pressure and heart rate variability

Blood pressure variability results are shown in Table 2 and HRV results are shown in Table 3. As can be seen, none of the components in either group changed significantly during the experiment.

**Table 2:** Short-term spectral analysis of blood pressure variability (BPV) results

	Control group				Experimental group			
	BL	SR	REC	*p*	BL	SR	REC	*p*
Total power (mmHg^2^)	24.2 ± 1.3	29.4 ± 5.3	33.6 ± 11	*F* = 0.346, *p* = .743	32.2 ± 6.3	61.2 ± 14	55.8 ± 7.7	*F* = 2.80, *p* = .083
ULF power (mmHg^2^)	1.9 ± 1.2	1.1 ± 0.70	1.7 ± 1.5	*F* = 1.15, *p* = .465	3.1 ± 0.60	7.2 ± 2.6	6.1 ± 1.8	*F* = 1.19, *p* = .322
VLF power (mmHg^2^)	15.6 ± 1.3	20.5 ± 2.1	22.2 ± 5.4	*F* = 0.709, *p* = .585	16.2 ± 4.7	40.5 ± 11	35.4 ± 6.6	*F* = 3.38, *p* = .053
LF power (mmHg^2^)	4.4 ± 2.3	6.3 ± 3.3	7.9 ± 5.8	*F* = 0.150, *p* = .869	10.4 ± 2.3	10.2 ± 1.9	10.1 ± 1.3	*F* = 0.010, *p* = .990
HF power (mmHg^2^)	2.4 ± 1.4	1.6 ± 0.80	1.9 ± 0.90	*F* = 1.58, *p* = .388	2.5 ± 0.75	3.3 ± 0.59	4.1 ± 1.0	*F* = 1.32, *p* = .287
LF/HF power (%)	196 ± 20	688 ± 565	354 ± 138	*F* = 0.464, *p* = .683	713 ± 212	370 ± 67	501 ± 147	*F* = 1.24, *p* = .309
Normalized LF power	0.66 ± 0.03	0.74 ± 0.19	0.76 ± 0.07	*F* = 0.156, *p* = .865	0.75 ± 0.06	0.74 ± 0.04	0.71 ± 0.05	*F* = 0.278, *p* = .760
Normalized HF power	0.34 ± 0.02	0.26 ± 0.19	0.24 ± 0.07	*F* = 0.149, *p* = .870	0.25 ± 0.06	0.26 ± 0.03	0.29 ± 0.05	*F* = 0.264, *p* = .770

Values are means ± SE. *F* and *p* values indicate results of repeated measurements ANOVA. For abbreviations, refer to the text

**Table 3:** Short-term spectral analysis of heart rate variability (HRV) results

	Control group				Experimental group			
	BL	SR	REC	*p*	BL	SR	REC	*p*
Total power (ms^2^)	3638 ± 770	4352 ± 2520	3309 ± 1098	*F* = 0.328, *p* = .753	4592 ± 945	5874 ± 2016	3995 ± 1331	*F* = 0.444, *p* = .647
ULF power (ms^2^)	208 ± 12	356 ± 335	134 ± 86	*F* = 0.446, *p* = .692	202 ± 65	437 ± 301	113 ± 22	*F* = 0.927, *p* = .411
VLF power (ms^2^)	955 ± 117	918 ± 537	779 ± 294	*F* = 0.195, *p* = .837	739 ± 138	1727 ± 639	720 ± 121	*F* = 2.57, *p* = .100
LF power (ms^2^)	881 ± 171	1200 ± 658	863 ± 153	*F* = 0.161, *p* = .862	2352 ± 813	1839 ± 604	1445 ± 531	*F* = 0.519, *p* = .602
HF power (ms^2^)	1581 ± 805	1842 ± 962	1521 ± 866	*F* = 4.66, *p* = .177	1286 ± 459	1837 ± 596	1695 ± 702	*F* = 0.287, *p* = .753
LF/HF power (%)	82.6 ± 52.9	63.9 ± 2.4	92.3 ± 62.6	*F* = 0.169, *p* = .855	260 ± 89.2	122 ± 16.2	115 ± 23.8	*F* = 2.32, *p* = .122
Normalized LF power	0.40 ± 0.17	0.39 ± 0.01	0.42 ± 0.19	*F* = 0.020, *p* = .981	0.56 ± 0.07	0.52 ± 0.03	0.49 ± 0.04	*F* = 0.636, *p* = .539
Normalized HF power	0.60 ± 0.17	0.60 ± 0.01	0.58 ± 0.19	*F* = 0.014, *p* = .987	0.44 ± 0.07	0.47 ± 0.03	0.51 ± 0.04	*F* = 0.636, *p* = .539
BRS (ms/mmHg)	22.5 ± 9.2	18.8 ± 2.5	20.8 ± 11	*F* = 0.176, *p* = .851	16.0 ± 1.9	15.3 ± 1.7	12.9 ± 1.7	*F* = 1.48, *p* = .249

Values are means ± SE. *F* and *p* values indicate results of repeated measurements ANOVA. For abbreviations, refer to the text

### Whole day heart rate variability

**Fig. 3:**
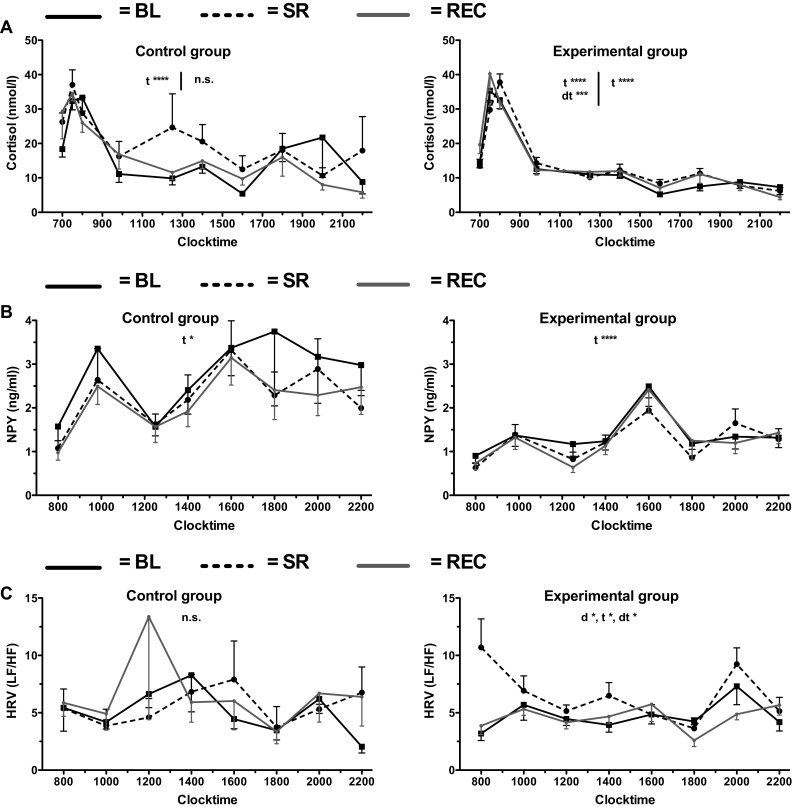
Cortisol (**a**), NPY (**b**), and whole day HRV (**c**) in both groups. Means ± SEM are plotted. Repeated measures ANOVA results are indicated with *t* (main effect of time), *d* (main effect of experimental day), and *dt* (interaction between *d* and *t*). *p < .05, **p < .01, ***p < .005, ****p < .001

The LF/HF power ratio remained unaffected in CON, whereas in EXP it differed between the profile days [*F*(2,22) = 5.64, *p* = .011], with lowest at BL (4.6 ± 0.4) and peaking at SR (6.0 ± 0.6; Fig. 3c). In addition, a slight effect of time of day was observed [*F*(7,77) = 2.16, *p* = .047]. The lowest levels were observed at 1800 hours (3.5 ± 0.4) and peak levels at 2000 hours (6.7 ± 0.9). Finally, there was a slight interaction between day and time [*F*(14,154) = 1.76, *p* = .049].

### Salivary neuropeptide Y levels

The salivary NPY levels did not differ between the three profile days in either group. The levels did, however, change with time in both CON [*F*(7, 42) = 3.12, *p* = .010] and EXP [*F*(7, 91) = 10.70, *p* < .001]. In CON, the levels ranged from 1.2 ± 0.18 at 0800 hours to 3.3 ± 0.60 at 1600 hours and in EXP it ranged from 0.74 ± 0.12 at 0800 hours to 2.3 ± 0.41 at 1600 hours.

### Salivary cortisol levels

For the morning values, a main effect of time was observed in both CON [*F*(4, 24) = 25.00, *p* < .001] and EXP [*F*(4, 48) = 75.27, *p* < .001]. In CON, salivary cortisol values peaked at 0730 hours (34.6 ± 2.66 nmol/L) and were lowest at 0950 hours (14.7 ± 3.10 nmol/L). In EXP, the cortisol values also peaked at 0730 hours (35.8 ± 2.67 nmol/L) and were lowest at 1230 hours (11.0 ± 1.07 nmol/L). Moreover, in EXP, an interaction was observed between day and time [*F*(8, 96) = 3.12, *p* = .004], indicating a phase shift. Indeed, the peak shifted forward after SR compared to BL, from 0730 to 0800 hours, and subsequently back to 0730 hours again after REC.

Afternoon cortisol values showed no effects in CON, and a main effect of only time in EXP [*F*(4, 48) = 7.41, *p* < .001], where cortisol values declined from 11.6 ± 1.1 nmol/L at 1400 hours to 5.8 ± 1.1 nmol/L at 2200 hours.

## Discussion

Long-term sleep restriction is becoming an increasingly common phenomenon in modern industrialized societies, due to, for instance, increasing work demands and increasing access to electronic entertainment [9, 48, 49]. Hitherto, however, few studies have investigated the effects of prolonged sleep restriction on parameters related to the integrated stress response. Moreover, the results of these earlier studies have been inconsistent [37–40]. The novelty of the present study is the usage of a design that reflects accumulating sleep restriction during five long working days followed by 2 days of weekend recovery sleep, adding thereby to the ecological validaty of these earlier results.

The main results of this study show that heart rate increased after 5 days of restricted sleep, and also after the 2 days recovery period. Blood pressure, however, remained unaffected. Since both blood pressure and heart rate are regulated by the autonomic sympatho-adrenal system, the observed differences in those parameters may seem unexpected. Heart rate, however, changes more acutely than blood pressure, and healthy blood vessels respond by dilation to prevent a rise in blood pressure. Hence, the observed absence of an increase in blood pressure is indicative of a good initial vascular health in our study population and does not mean that the autonomic activity did remain unaffected. In fact, the increase in heart rate suggests increased sympathetic activity. Therefore, if the period of sleep restriction would have been extended to several weeks, it cannot be ruled out that this eventually would have resulted in increased blood pressure as well, such as for example observed by Tochikubo et al. in a healthy population [40] or Lusardi et al. in a hypertensive population [50].

The study population in the current study was young and healthy. Therefore, conclusions as to how a less healthy or older population would have been affected cannot be drawn. It is not unlikely, though, that the effects in that case would have been more serious, due to, for instance, a reduction in cardiovascular flexibility [51]. Elevated heart rate in itself, however, is already a major risk factor for the development of cardiovascular diseases, including hypertension and atherosclerosis [52].

Another parameter that reflects the activity of the autonomic sympatho-adrenal system is heart rate variability. The high-frequency (HF) component, ranging from 0.15 to 0.4 Hz, represents the frequency of respiration and is mediated by the parasympathetic branch of the autonomic nervous system. The low-frequency (LF) component (0.04–0.15 Hz) reflects inputs from both the sympathetic and the parasympathetic branch of the ANS. As a consequence, the LF/HF power ratio is an acceptable and widely used measure of sympathetic modulation [45, 53], despite that some studies do not agree with this interpretation [54]. Although short-term heart rate and blood pressure variability remained unaffected in the present study, the LF/HF power ratio over the whole day was increased after five nights of sleep restriction. This is in line with the observed heart rate increase and also indicative of increased activity in the autonomic sympatho-adrenal system after a period of short sleep. Moreover, it is in line with results that were obtained by Zhong et al. in an acute sleep deprivation setting [36] as well as those of Dettoni and colleagues in a setting of less than 5 h sleep for a period of five nights [39].

Cortisol and neuropeptide Y are well-known stress hormones that usually elevate in a similar way in response to acute stress [55]. It has also been suggested that NPY promotes sleep, but the interaction between cortisol and NPY remains unclear [56, 57].

In the present study, they indeed responded in a similar way to sleep restriction, which is their levels were not affected. Particularly regarding salivary cortisol, which is a widely studied stress indicator, this is in line with some studies [37], but not in line with other studies of partial sleep restriction [38]. It has been hypothesized and shown that sleep disturbing and/or restricting protocols can result in lowered morning, but higher evening levels of cortisol, i.e., decreased amplitude of the rhythm [58]. Despite our separate analyses of morning values and afternoon/evening values, we did not observe either of those effects. This may, of course, indicate that 5 days of restricted sleep is insufficient to elicit a clear stress response and that a clear response might have followed if sleep restriction was prolonged further. Another interesting explanation might be in the timing of the 4 h time in bed that participants were allowed. Most studies place this period right in the middle of the habitual time in bed, thus delaying bedtime with 2 h and advancing wake up time with 2 h, e.g., [38, 59]. Although such studies may have valid reasons to do so, it does result in wake up times that are in or very near the circadian trough, namely at 0500 hours. It might be that waking up per se at such an inconvenient time, rather than at a more usual 0700 hours, results in additional stress responses in participants. Hence, in such studies, not only sleep is restricted, but also wake up time is advanced to what arguably is a very uncomfortable time to rise. Therefore, we can certainly not rule out that the cortisol results of the present study would have been more in line with previous work (i.e., showing a decreased amplitude), if we had shifted the time in bed period 2 h back to 0100–0500 hours instead of 0300–0700 hours. Indeed, it has been shown in several studies that early awakenings per se result in cortisol elevation throughout the entire day [60, 61].

In conclusion, we showed that the effects of 1 week of sleep restriction on the autonomic system in a young and healthy study population are limited. Both heart rate and whole day heart rate variability were increased in a manner suggesting increased sympathetic activity. Those are also the first parameters in which such an increase would be noticeable. Other, possibly more serious, changes, such as those in blood pressure, may simply require more time to develop. Of special interest may be the discrepancy in cortisol responses between the present study and some of the previous studies, which might be solely attributable to the manner in which time in bed was scheduled. Finally, the possibility cannot be ruled out that more severe consequences would have been observed in an older and/or less healthy study population due to reduced cardiovascular flexibility [51] as well as when the current study would have been prolonged reflecting several working weeks in a row, thereby better reflecting the prolonged effects of sleep restriction.
